# ROCK Inhibitor Y27632 Induced Morphological Shift and Enhanced Neurite Outgrowth-Promoting Property of Olfactory Ensheathing Cells via YAP-Dependent Up-Regulation of L1-CAM

**DOI:** 10.3389/fncel.2018.00489

**Published:** 2018-12-11

**Authors:** Yijian Li, Shujia Huo, Yajie Fang, Ting Zou, Xianliang Gu, Qin Tao, Haiwei Xu

**Affiliations:** ^1^Southwest Hospital, Southwest Eye Hospital, Third Military Medical University (Army Medical University), Chongqing, China; ^2^Key Lab of Visual Damage and Regeneration & Restoration of Chongqing, Chongqing, China

**Keywords:** olfactory ensheathing cells, morphology, ROCK, YAP, neurite outgrowth

## Abstract

Olfactory ensheathing cells (OECs) are heterogeneous in morphology, antigenic profiles and functions, and these OEC subpopulations have shown different outcomes following OEC transplantation for central nervous system (CNS) injuries. Morphologically, OECs are divided into two subpopulations, process-bearing (Schwann cells-like) and flattened (astrocytes-like) OECs, which could switch between each other and are affected by extracellular and intracellular factors. However, neither the relationship between the morphology and function of OECs nor their molecular mechanisms have been clarified. In the present study, we first investigated morphological and functional differences of OECs under different cytokine exposure conditions. It demonstrated that OECs mainly displayed a process-bearing shape under pro-inflammatory conditions (lipopolysaccharide, LPS), while they displayed a flattened shape under anti-inflammatory conditions [interleukin-4 (IL-4) and transforming growth factor-β1 (TGF-β1)]. The morphological changes were partially reversible and the Rho-associated coiled-coil-containing protein kinase (ROCK)/F-actin pathway was involved. Functionally, process-bearing OECs under pro-inflammatory conditions showed increased cellular metabolic activity and a higher migratory rate when compared with flattened OECs under anti-inflammatory conditions and significantly promoted neurite outgrowth and extension. Remarkably, the morphological shift towards process-bearing OECs induced by ROCK inhibitor Y27632 enhanced the neurite outgrowth-promoting property of OECs. Furthermore, as the downstream of the ROCK pathway, transcriptional co-activator Yes-associated protein (YAP) mediated morphological shift and enhanced the neurite outgrowth-promoting property of OECs through upregulating the expression of the neural adhesion molecule L1-CAM. Our data provided evidence that OECs with specific shapes correspond to specific functional phenotypes and opened new insights into the potential combination of OECs and small-molecule ROCK inhibitors for the regeneration of CNS injuries.

## Introduction

In the olfactory system, olfactory ensheathing cells (OECs), also known as olfactory ensheathing glia or olfactory Schwann cells, project from the olfactory epithelium (OE) to the olfactory bulb (OB) in the central nervous system (CNS) and envelop several olfactory receptor neuron (ORN) axons (Kott et al., 1994; Su and He, 2010). During the development of the olfactory sensory system, OECs release diffusible factors, which regulate the proliferation and differentiation of neural progenitor cells through activating the Notch signaling pathway (Zhang et al., 2008). In addition, OECs are thought to facilitate the outgrowth of ORN axons and support their extension not only by providing favorable growth factors but also by direct cellular contact (Kafitz and Greer, 1999; Li et al., 2005; Wei et al., 2008; Liu Q. et al., 2010). Transplanted OECs have been shown to promote axonal regeneration, neuronal tissue reconstruction and functional recovery in CNS lesions through secretion of neurotrophic factors, production of cell adhesion molecules, rapid removal of dead neurons and their capacity to orchestrate axonal extension across CNS lesion regions (Raisman, 2001; Radtke et al., 2004; Raisman and Li, 2007; Yang et al., 2015). Thus, OECs have emerged to be a remarkable candidate for stimulating regeneration in CNS injuries and neurodegenerative diseases (Gómez et al., 2018).

OECs are heterogeneous, which is attributed to their differences in age, sources of donor tissue, methods of isolation and culture conditions. Several subpopulations of OECs have been recognized based on their anatomical locations, functions and antigenic profiles (Richter et al., 2005; Windus et al., 2010). Furthermore, these OEC subpopulations have shown different biological functions and affected outcomes following transplantation (Richter et al., 2005; Windus et al., 2010). Morphologically, cultured OECs have two subpopulations, process-bearing (Schwann cells-like) OECs with a long fusiform bipolar morphology and flattened (astrocytes-like) OECs with orientated processes and a flat sheet-like morphology (Huang et al., 2008), which can switch between each other and are affected by extracellular [extracellular matrix (ECM), media] and intracellular (e.g., cAMP) factors (Doucette and Devon, 1995; Vincent et al., 2003; Vukovic et al., 2009). Furthermore, transplanted OECs also displayed morphological plasticity (Li et al., 1998). *In vitro*, process-bearing OECs abundantly expressed p75NTR, S100β and weakly expressed GFAP, whereas flattened OECs lacked p75NTR but expressed S100β and mostly bright GFAP (Pixley, 1992). One previous study showed that p75NTR-positive OECs were more effective in promoting neurite regrowth when co-cultured with adult rat retinal ganglion cells (RGCs; Kumar et al., 2005). It appears that morphological plasticity and differential expressions of markers by OECs are more likely a reflection of their functional plasticity. To be in line with this notion, several studies demonstrated that morphological phenotypes of OECs exhibited different migratory properties (Huang et al., 2008, 2011a; Wang and Huang, 2012). All these studies make it clear that OECs are a single but malleable phenotype with extensive morphological and functional plasticity depending on the environmental stimuli (Vincent et al., 2005; Huang et al., 2008).

The Rho GTPases are critical regulators that control assembly, disassembly and dynamic rearrangements of actin and microtubule cytoskeletons. Besides cytoskeleton and cell morphology, Rho GTPases also regulate a series of cellular functions, such as cell adhesion, endocytosis, migration and differentiation (Burridge and Wennerberg, 2004). One of the best-characterized effectors of Rho GTPases is Rho-associated coiled-coil-containing protein kinase (ROCK). ROCK mediates morphological switches through regulating its substrates, such as myosin light chain (MLC), MLC phosphatase (MLCP) and LIM kinase 1/2 (LIMK1/2; Mueller et al., 2005). Furthermore, transcriptional co-activator Yes-associated protein (YAP) has emerged as a hub that integrates diverse stimuli including mechanical and cytoskeletal cues, cell adhesion and ECM stiffness, and regulated tissue development and regeneration after injury (Yang and Xu, 2011). The activity of YAP appears to be regulated mainly by phosphorylation. Unphosphorylated YAP is located in the nucleus, where it functions as co-factor of transcription by interactions with TEAD, Runx or SMAD. When phosphorylated at Ser127 site, YAP was sequestered in the cytoplasm by 14-3-3σ and gradually degradated (Yu et al., 2015).

Our previous study found that transforming growth factor β1 (TGF-β1) induced flattened OECs with increased phagocytic activity (Li et al., 2017). In the present study, we further investigated the morphological and functional transformation of OECs and their influence on neurite outgrowth and extension. Furthermore, the molecular mechanisms of morphological shift and the enhanced neurite outgrowth-promoting property of OECs, including the ROCK/YAP/L1-CAM pathway, were also explored.

## Materials and Methods

### Ethical Statements

All animals in this study were housed and treated in strict accordance with the recommended NIH guidelines for animal care and safety and were approved by the Ethics Committee for Animal Research at the Third Military Medical University (Army Medical University).

### Regents and Antibodies

Lipopolysaccharide (LPS; Solarbio, L8880), IL-4 (Sino Biological, 11846-HNAE), TGF-β1 (Sino Biological, 10804-HNAC) and BrdU (Sigma-B5002) were dissolved in sterile water with 1,000× stock concentration. Y27632 (Peprotech, 1293823) and verteporfin (MedChem Express, CL318952) were dissolved in DMSO with 1,000× stock concentration. For regent treatments, corresponding vehicle was used as the control.

The following primary antibodies were used in immunocytochemical (ICC) staining and Western blotting (WB). S100 (rabbit, Sigma, SAB5500172; ICC, 1:800), GFAP (mouse, Abcam, ab10062; ICC, 1:600), p-MLC (rabbit, Abcam, ab157747; WB, 1:2,000), Ki67 (mouse, Abcam, ab8191; ICC, 1:600), BrdU (mouse, Abcam, ab8152; ICC, 1:500), Tuj1 (mouse, Abcam, ab78078; ICC, 1:600), Tau (mouse, Abcam, ab80579; ICC, 1:400), p75NTR (mouse, Abcam, ab3125; ICC, 1:400), YAP1 (rabbit, Abcam, ab39361; ICC, 1:600; WB: 1:2,000), YAP1 (phospho S127; rabbit, Abcam, ab76252; WB, 1:2,000), CTGF (mouse, Santa Cruz, sc-365970; WB, 1:3,000), integrin beta 5 (rabbit, Abcam, ab15459; WB, 1:1,000), FAK (rabbit, Abcam, ab40794; WB, 1:3,000), FAK (phospho Y397; rabbit, Abcam, ab81298; WB, 1:2,000), L1-CAM (mouse, Abcam, ab24345; WB, 1:1500), GAPDH (rabbit, Abcam, ab181602; WB, 1:3,000) and β-actin (mouse, Boster, BM0627; WB, 1:4,000). For ICC, Fluor-488- or 568-conjugated anti-mouse or rabbit immunoglobulin-G (IgG; Invitrogen, Carlsbad, CA, USA; A-11008, A32723, A-11004 and A-11011) diluted 1:600 in PBS were used. For WB, horseradish peroxidase (HRP)-conjugated secondary antibodies (Abcam, ab6789 and ab205718; 1:3,000) were used. Primary L1-CAM antibody (mouse, R&D Systems, MAB5674) was used for blocking L1-CAM functionally.

### Primary Culture of OECs and Purification

Primary OECs were prepared from the OB of adult Long Evans rats and purified by differential cell adhesiveness as described previously (Liu Y. et al., 2010; Li et al., 2017). Briefly, after meninges were removed from the OB, the olfactory nerve layer (ONL) was peeled away from the glomerular and deeper layers of the OB. Tissues were chopped and digested in 0.25% trypsin (Sigma) for 15 min. After ending digestion by DMEM/F-12 (Hyclone) containing 10% fetal bovine serum (FBS, Gibco), these chopped tissues were further mechanically dissociated into single cell suspension. The cell suspension was plated on uncoated 6 cm culture dishes (NEST) twice, each for 24 h. These non-adhesive cells were collected, spun down, resuspended in culture medium and seeded onto poly-D-lysine (PDL, 0.1 mg/mL, Sigma) pre-coated 6-well plates.

The morphological classification of OECs has been described previously (Huang et al., 2008). Briefly, process-bearing OECs were defined as those with two processes and a long fusiform bipolar morphology. Flattened OECs were defined as those with orientated processes and flat sheet-like morphology. Flattened OECs include two subtypes according to the location of the nucleus. Flattened type 1 OECs exhibit a fan-like shape with a nucleus lying at the edge of cytoplasm. Flattened type 2 OECs exhibit a round shape with a nucleus lying at the center of cytoplasm.

### Cortical Neurons and OECs Co-cultures

Purified OECs were seeded on glass slides and pre-treated with 10 μg/mL LPS, 10 ng/mL IL-4, 10 ng/mL TGF-β1 or 10μM Y27632 in DMEM/F12 containing 3% FBS for 24 h. Cortical neurons were cultured from embryonic day 18 (E18) Long Evans rats as previously described (Dietrich et al., 2003; Su et al., 2013). Briefly, brains were washed using ice-cold Hanks’ balanced salt solution (HBSS, Sigma). Cortical tissues were demembrated, chopped into small pieces, and incubated with 15 U/mL papain (Sigma) for 10 min. Then, cells were gently triturated using fire-polished Pasteur pipettes. Neurons were resuspended in DMEM/F12 containing 15% FBS after being centrifuged for 5 min at 300 *g*. After large fragments were allowed to settle for 10 min, neurons in supernatant were seeded onto OEC cultures. After 4 h, the culture medium was changed to reduce cell fragments. The co-cultures of cortical neurons and OECs were further cultured in DMEM/F12 containing 3% FBS for 3 days and subsequently examined.

### Time-Lapse Imaging of OECs

Purified OECs were seeded onto PDL-coated 6-well plates at a low density (5,000 cells per well). The back of the 6-well plate was lined to make sure of the locations of OECs that were observed during the time-lapse imaging. The isolated cells with typical OEC morphology were selected for time-lapse imaging. The purified OECs were treated with 10 μg/mL LPS, 10 ng/mL IL-4 and 10 ng/mL TGF-β1 in DMEM/F12 containing 3% FBS. The morphological changes of OECs were recorded with a CCD camera attached to the inverted phase microscope (LEICA DM IRE2). Two shape factors were used to describe the morphological properties of OECs with ImageJ software. The average process length was the average of leading and trailing processes of OECs. The circularity was a function of the perimeter *P* and the area *A*:

$$\text{Circularity = 4}\pi A\text{/}P^{2}$$

The circularity of a circle is 1 and much less than 1 for a process-bearing morphology.

### Immunocytochemical Staining

OECs on glass slides were fixed with 4% paraformaldehyde (PFA) and permeabilized with 0.3% TritonX-100. For BrdU staining, OECs were labeled with BrdU (10 μM) for 24 h, fixed in cold methanol and DNA was denatured with 2 mol/L HCl. After being blocked with 3% bovine serum albumin (BSA), OECs were incubated with primary antibodies at 4°C for 16 h. After washes with PBS, OECs were incubated with Fluor-488- or 568-conjugated anti-mouse or rabbit IgG (Invitrogen) diluted 1:600 in PBS at 37°C for 1 h. Cell nuclei were counterstained with DAPI (Beyotime). For F-actin staining, OECs were incubated with fluorescein-conjugated phalloidin (10 ng/mL; Sigma, P5282) for 30 min. Pictures were taken with a fluorescence microscope (BX51; Olympus) or confocal microscope (FV1000; Olympus), and analyzed with ImageJ software.

### *In vitro* Scratch Assay

An *in vitro* scratch assay was used to measure OECs’ migrating ability as well as neurite outgrowth and extension (Liang et al., 2007; Vukovic et al., 2009). For the migrating ability assay, purified OECs were seeded in 12-well plates and cultured for 24 h. Scratches across the center of the wells were generated with sterile 1 mL pipette tips using a ruler as a guide. Detached cells were removed by several washes. OECs were treated with 10 μg/mL LPS, 10 ng/mL IL-4, or 10 ng/mL TGF-β1. The images from the same field of view were captured at 24 and 48 h after the initial scratch injury. The images of the initial scratches were also captured as the initial control, which was represented as dotted lines. The number of OECs that migrated into scratches per field (50×) was measured and averaged at each time point with ImageJ software. To examine neurite outgrowth and extension, neurons cultured on astrocytes were scratched and detached cells were removed by several washes. After OECs were seeded with or without Y27632, extended neurites across scratches were visualized by Tau staining.

### Matrigel Drop Migration Assay

The matrigel drop migration assay was performed according to an agarose drop assay that has been described previously (Ogier et al., 2006; Gueye et al., 2011). Briefly, purified OECs were resuspended in a matrigel basement membrane matrix (BD Bioscience). 0.5 μL matrigel drops containing OECs were seeded onto 6-well plates and placed at 37°C to allow the matrigel to solidify. DMEM/F12 was added to cover the solidified matrigel drop. Then, OECs were treated with 10 μg/mL LPS, 10 ng/mL IL-4, or 10 ng/mL TGF-β1 in DMEM/F12 containing 3% FBS. The migration of OECs was carefully observed and photographed through a LEICA DM IRE2 inverted phase microscope. The cell migration distance index and migration area index were analyzed using ImageJ software. The matrigel drop area and the area of migrating cells were measured and the migration area index was represented as the ratio of the migrating cells area to the matrigel drop area. The longest migration distances out of the matrigel drops in each quadrant were measured, and the migration distance index was represented as the ratio of average cell migration distance to the radius of the matrigel drop.

### The Trans-Well Chamber Migration Assay

The trans-well migration assay was used to analyze the migratory response of purified OECs toward neurons (Su et al., 2009). The primary cortical neurons were seeded and cultured in 12-well plates. Purified OECs were counted and seeded into the upper chamber of trans-well inserts with a polyethylene terephtalate filter membrane with 8 μm-diameter pores. After pre-treatment with LPS, IL-4 and TGF-β1 in DMEM/F12 containing 3% FBS for 12 h, the trans-well inserts with OECs were transferred to the 12-well plates culturing primary cortical neurons. After co-culture for 48 h, the non-migratory OECs on the upper membrane surface were removed with cotton swabs, while the migrated OECs on the underside surface of the membrane were fixed with 4% PFA and stained with DAPI. For quantitative assessment, the number of nuclei stained migrating OECs was counted at five random fields per filter membrane in three independent experiments.

### Analysis of Neurite Outgrowth and Extension

To investigate neurite outgrowth and extension, the number of neurites, branches and the length of neurites were measured (Witheford et al., 2013). Briefly, four randomly-chosen fields in each glass slide were photographed through a fluorescent microscope. All Tuj-1 immunofluorescent cells were selected to measure the number of neurites, number of branches and length of neurites. At least 20 neurons in each glass slide were assessed for each group. The numbers of primary, secondary and tertiary neurites were assessed. The primary neurite was defined as the neurite that extended from the body of neurons. The secondary neurite was defined as the neurite that extended from the primary neurite, and tertiary neurite was defined as the neurite that extended from the secondary neurite. The number of neurites was the number of primary neurites. The number of branches was the total number of secondary and tertiary neurites. Neurite length was defined as the length between the body and the farthest tip of the neurite.

### Western Blotting

Protein samples of OECs were reconstituted in a sample buffer and denatured by boiling at 100°C for 5 min. Protein lysates were loaded on SDS-PAGE for electrophoresis and electrophoretically transferred onto a methanol-activated polyvinylidene difluoride (PVDF) membrane (Millipore). Non-specific protein was blocked by incubation with 5% BSA diluted in Tris-buffered saline (TBS) containing 0.05% Tween-20 (TBST) for 2 h. The membrane was probed by primary antibodies at 4°C for 16 h and subsequently recognized by incubation with HRP-conjugated secondary antibodies for 2 h at room temperature. The immuno-reactive bands were presented by reacting with chemiluminescence reagents (ECL, Beyotime).

### siRNA Knockdown

YAP siRNAs and scrambled siRNA controls were obtained from OriGene Technologies (China). OECs were seeded in 6-well plates, incubated overnight to a density of 70% and transfected using Lipofectamine 2000 (Thermo Fisher Scientific Waltham, MA, USA) for 72 h according to the manufacturer’s instructions. Afterwards, the medium was replaced with a fresh medium for subsequent protein analysis.

### Statistical Analysis

All data were represented as mean ± SD from at least three independent experiments. Statistical analysis was performed using Student’s *t*-test, one-way analysis of variance (ANOVA) or two-way ANOVA with Dunnett’s post test and χ^2^ test. Statistical significance was defined as *P < 0.05, **P < 0.01, ***P < 0.001, ****P < 0.0001.

## Results

### Morphological Changes of OECs Under Different Inflammatory Conditions

**Figure 1:**
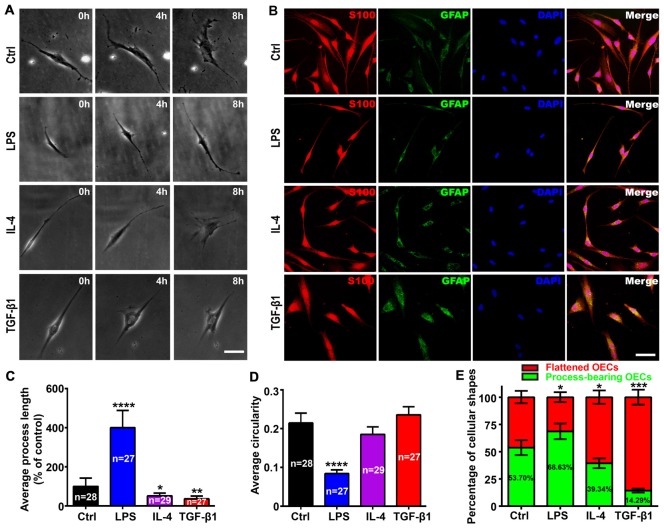
Morphological changes of olfactory ensheathing cells (OECs) under pro- and anti-inflammatory conditions. **(A)** Phase contrast images of single purified OEC treated with 10 μg/mL LPS, 10 ng/mL IL-4 and 10 ng/mL TGF-β1 at 0, 4 and 8 h. **(B)** Immunostaining images for S100 and GFAP of purified OECs treated with 10 μg/mL LPS, 10 ng/mL IL-4 and 10 ng/mL TGF-β1 for 24 h. Cell nuclei were counterstained with DAPI. **(C,D)** Histograms show average process length and circularity for LPS, IL-4 and TGF-β1-treated purified OECs for 24 h. Values in the histogram represent the counted number of OECs. **(E)** Histogram shows percentage of flattened and process-bearing OECs for LPS, IL-4 and TGF-β1-treated purified OECs for 24 h. Values in the histogram represent the percentage of process-bearing OECs. Data are represented as mean ± SD, *n* = 3 experiments. Scale bar, 25 μm. *P < 0.05, **P < 0.01, ***P < 0.001, ****P < 0.0001; one-way ANOVA with Dunnett’s post test **(C, D**) and χ^2^ (and Fisher’s exact) test **(E)**, comparison with control.

According to the criteria of process-bearing and flattened OECs (Huang et al., 2008), we first analyzed the effects of pro-inflammatory (LPS) and anti-inflammatory (IL-4 and TGF-β1) conditions on the cell morphology of OEC using low-density OECs cultured on PDL-coated plates by time-lapse images. OECs under control conditions (DMEM/F12 containing 3% FBS) maintained a flattened appearance with short processes. When under pro-inflammatory conditions, OECs changed into a process-bearing shape with extremely long processes (Figure 1A). However, when under anti-inflammatory conditions, OECs changed to a flattened shape and showed a flat sheet-like shape within 8 h (Figure 1A). The morphological changes of OECs cultured at high-density were similar to low-density cultures (Figure 1B). The average process length of OECs increased and the circularity decreased under pro-inflammatory conditions, indicating an increased degree of cell elongation of OECs. In contrast, the average process length of OECs decreased under anti-inflammatory conditions (Figures 1C,D). Furthermore, pro-inflammatory conditions resulted in an increased proportion of process-bearing OECs, whereas anti-inflammatory conditions caused an increased proportion of flattened OECs (Figure 1E).

As the ECM modulated cell morphology (Tisay and Key, 1999; Vukovic et al., 2009), we next analyzed morphological changes of OECs on different substrates under pro- and anti-inflammatory conditions. Similar results were observed when OECs were grown on gelatin or laminin-coated surfaces (Supplementary Figure S1). Taken together, it strongly suggested that pro- and anti-inflammatory culture conditions were sufficient to induce morphological changes of OECs.

### Partially Reversible Changes of OECs Shapes Under Pro- and Anti-inflammatory Conditions

**Figure 2:**
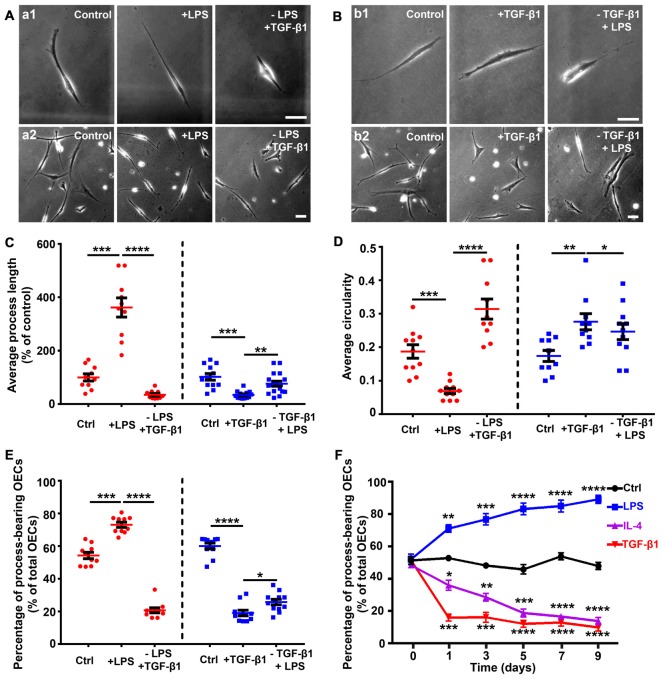
Partially reversible changes and maintenance of OEC morphology under pro- and anti-inflammatory conditions. **(A)** Replacement of LPS (10 μg/mL, 24 h) with 10 ng/mL TGF-β1 caused process-bearing OECs to change to flattened shape after 12 h. Single-cell analysis (upper panel) and high-density OECs (lower panel). **(B)** Replacement of TGF-β1 (10 ng/mL, 24 h) with 10 μg/mL LPS caused flattened OECs to change to process-bearing shape with short process after 36 h. Single-cell analysis (upper panel) and high-density OECs (lower panel). **(C,D)** Quantification of average process length and circularity of purified OECs with the changes of pro- and anti-inflammatory cytokines. **(E)** Percentage of process-bearing OECs with changes of pro- and anti-inflammatory cytokines. **(F)** OECs maintained process-bearing or flattened morphology under constant pro- or anti-inflammatory conditions. Data are represented as mean ± SD from at least three experiments. *P < 0.05, **P < 0.01, ***P < 0.001, ****P < 0.0001; two-tailed unpaired Student’s *t*-test **(C–E)**; two-way ANOVA with Dunnett’s post test **(F)**, comparison with control.

Reversible morphological changes have been reported between process-bearing and flattened OECs independent of any environmental stimuli (spontaneously) or induced by applying endothelin-1 or increasing the intracellular level of cAMP (Huang et al., 2008; van den Pol and Santarelli, 2003; Vincent et al., 2003). In the present study, OECs switched to a process-bearing shape within 24 h by adding LPS. When LPS was replaced with TGF-β1, process-bearing OECs returned to a flattened shape within 12 h (Figures 2A,C,D). Conversely, when TGF-β1 was replaced with LPS, flattened OECs partially returned to a process-bearing shape within 36 h (Figures 2B–D). LPS treatment increased the proportion of process-bearing OECs, and the proportion of process-bearing OECs significantly decreased by replacing LPS with TGF-β1 within 12 h. Interestingly, the proportion of process-bearing OECs could be partially reversed by replacing TGF-β1 with LPS within 36 h (Figure 2E). These results suggested that pro- and anti-inflammatory conditions induced partially reversible morphological changes of OECs. In addition, OECs maintained their process-bearing or flattened shape for at least 9 days under constant pro- or anti-inflammatory conditions (Figure 2F).

### ROCK/F-Actin Pathway Mediates Morphological Changes of OECs

**Figure 3:**
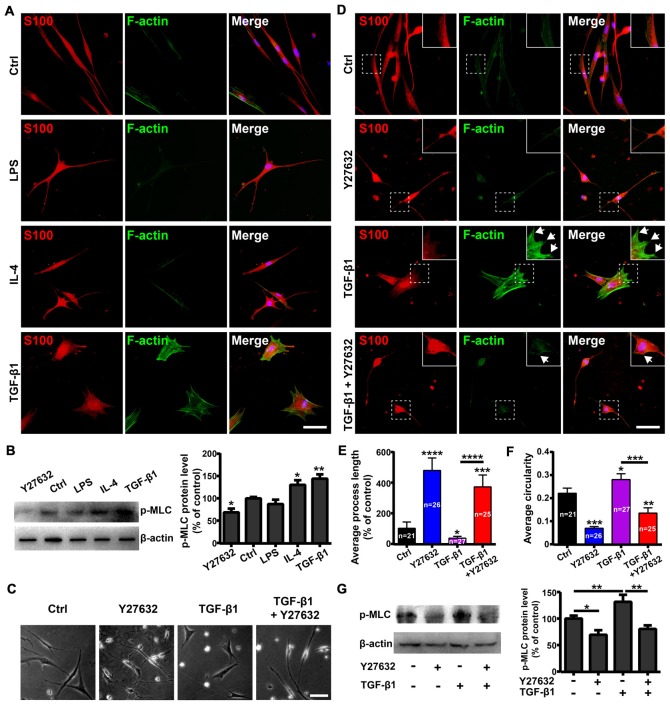
Rho-associatedcoiled-coil-containing protein kinase (ROCK)/F-actin pathway mediates morphological changes of OECs. **(A)** Fluorescein-conjugated phalloidin staining for F-actin of OECs under pro-inflammatory (LPS, 10 μg/mL) and anti-inflammatory (IL-4 and TGF-β1, 10 ng/mL) conditions for 24 h. **(B)** OECs were treated with vehicle, 10 μg/mL LPS, 10 ng/mL IL-4, 10 ng/mL TGF-β1 or 10 μM Y27632 for 24 h and performed Western blotting (WB) analysis for phosphorylated myosin light chain (p-MLC). **(C)** Y27632 (10 μM) inhibits morphological changes of OECs induced by TGF-β1 (10 ng/mL). **(D)** Y27632 (10 μM) inhibits formation of stress fibers and membrane ruffles of OECs induced by TGF-β1 (10 ng/mL). Arrowheads indicate the membrane ruffles. **(E,F)** Histograms show average process length and circularity of OECs under indicated conditions for 24 h. Values in the histogram represent the counted number of OECs. **(G)** WB analysis for p-MLC of OECs under indicated conditions for 24 h. All quantitative assessments of WB were normalized by β-actin. Data are represented as mean ± SD, *n* = 3 experiments. Scale bar, 50 μm. *P < 0.05, **P < 0.01, ***P < 0.001, ****P < 0.0001; one-way ANOVA with Dunnett’s post test **(B,E–G)**, comparison with control.

The Rho GTPases (including RhoA, Rac1 and Cdc42) play important roles in regulating cell morphology and the RhoA/ROCK/Myosin/F-actin pathway mediates the morphological plasticity of cultured OECs (Etienne-Manneville and Hall, 2002; Huang et al., 2011b). Thus, we evaluated whether morphological changes of OECs under pro- and anti-inflammatory conditions were attributed to different expression patterns of stress fibers by F-actin staining. OECs under pro-inflammatory conditions (LPS) formed few stress fibers. In contrast, OECs under anti-inflammatory conditions (IL-4 and TGF-β1) formed bundles of actin filaments at the edge and thick parallel bundles of actin filaments that spanned the entire cell body (Figure 3A). These results indicated that anti-inflammatory conditions induced morphological changes of OECs towards a flattened shape through cytoskeletal re-arrangements.

Next, we investigated whether the ROCK pathway mediated morphological changes of OECs. WB showed that LPS treatment decreased protein levels of phosphorylated MLC (p-MLC), one of the main substrates of ROCK. In contrast, TGF-β1 and IL-4 treatments increased p-MLC protein levels (Figure 3B), indicating that the ROCK pathway was involved in the morphological changes of OECs under pro- and anti-inflammatory conditions.

In addition, Y27632, a ROCK-specific inhibitor, promoted OECs to switch towards a process-bearing shape and markedly inhibited the formation of stress fibers and membrane ruffles induced by TGF-β1 (Figures 3C–F). Furthermore, Y27632 significantly decreased the p-MLC protein level and blocked morphological changes of OECs towards the flattened shape induced by TGF-β1 (Figures 3C–G). Taken together, these results suggested that the ROCK/F-actin pathway mediated TGF-β1-induced cytoskeletal re-arrangements and morphological changes of OECs.

### Influences of Pro- and Anti-inflammatory Conditions on Proliferation and Migration of OECs

The OECs’ morphology is associated with a specific functional phenotype (Vincent et al., 2005; Ekberg et al., 2012; Wang and Huang, 2012). Here, we explored the relationship between the morphological changes and functions of OECs under pro- and anti-inflammatory conditions. The proliferation of OECs was tested by Ki67 and BrdU staining. No significant influences on proliferation were observed when OECs were treated with LPS, IL-4 or TGF-β1 in comparison with the control (Supplementary Figures S2A–D). The average cell number of OECs showed no significant difference either (Supplementary Figure S2E). This suggested that pro- and anti-inflammatory conditions had no significant influences on the proliferation of OECs. However, the CCK-8 assay, detecting cellular metabolic activities of mitochondrial enzymes, showed significant difference between pro- and anti-inflammatory conditions, as pro-inflammatory conditions enhanced the cellular metabolic activity of OECs (Supplementary Figure S2F).

**Figure 4:**
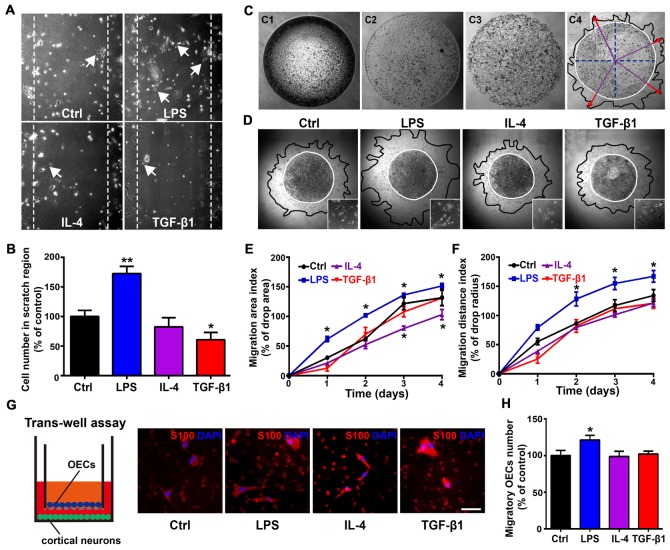
Differential migratory abilities of OECs under pro- and anti-inflammatory conditions. **(A,B)** OECs migrated into scratches under pro- or anti-inflammatory conditions at 48 h. Arrowheads indicate OECs migrating into scratches. Quantitative analysis of OEC numbers migrated into scratch regions. **(C)** Schematic of the matrigel drop migration assay. Matrigel drops containing OECs were seeded on plates and flooded by medium after solidification. OECs survived and migrated out of the matrigel drop. Migration area (black line) and migration distance (red arrowhead) are shown. **(D)** Images of OECs migrated out of matrigel drops under pro- or anti-inflammatory conditions at 72 h. The inserts show the shape of OECs that migrated out of matrigel drops under indicated conditions. **(E,F)** Quantification of OEC migration area index and migration distance index under pro- or anti-inflammatory conditions at 1, 2, 3 and 4 days. **(G,H)** OECs migrated through the filter membrane of trans-well insert with primary cortical neurons cultured on plates. Quantitative analysis of number of OECs migrated through the filter membrane of trans-well insert after 48 h. Scale bar, 50 μm. Data are represented as mean ± SD, *n* = 3 experiments. *P < 0.05, **P < 0.01; one-way ANOVA **(B,H)** and two-way ANOVA **(E, F**) with Dunnett’s post test, comparison with control.

As morphological phenotypes of OECs displayed differential migratory properties and process-bearing OECs were more motile than flattened OECs (Huang et al., 2008; Wang and Huang, 2012), we then examined whether OECs under pro- and anti-inflammatory conditions also displayed differential migratory activities. In an *in vitro* scratch assay, process-bearing OECs under pro-inflammatory conditions covered more regions of scratch gaps and more OECs migrated into scratch gaps (Figures 4A,B). In contrast, flattened OECs under anti-inflammatory conditions showed less OECs migrating into scratch gaps, indicating that process-bearing OECs under pro-inflammatory conditions are more motile than flattened OECs under anti-inflammatory conditions (Figures 4A,B). In the matrigel drop assay (Figure 4C), spontaneous and un-directional movements of OECs under pro- and anti-inflammatory conditions were examined. Process-bearing OECs under pro-inflammatory conditions covered more area and migrated longer distances than OECs under control and anti-inflammatory conditions (Figures 4D–F).

Previous studies showed that migration of OECs was directly related to the rate of olfactory axon growth (Chehrehasa et al., 2010; Windus et al., 2011), so we examined the directional movement of OECs towards neurons using a trans-well chamber migration assay. OECs that migrated through the insert filter membrane increased by 20% under pro-inflammatory conditions compared to those under control and anti-inflammatory conditions (Figures 4G,H). Thus, it showed that process-bearing OECs under pro-inflammatory conditions displayed stronger migratory activities than flattened OECs under anti-inflammatory conditions.

### Morphological Shift of OECs Under Pro- and Anti-inflammatory Conditions Affected Neurite Outgrowth and Extension

**Figure 5:**
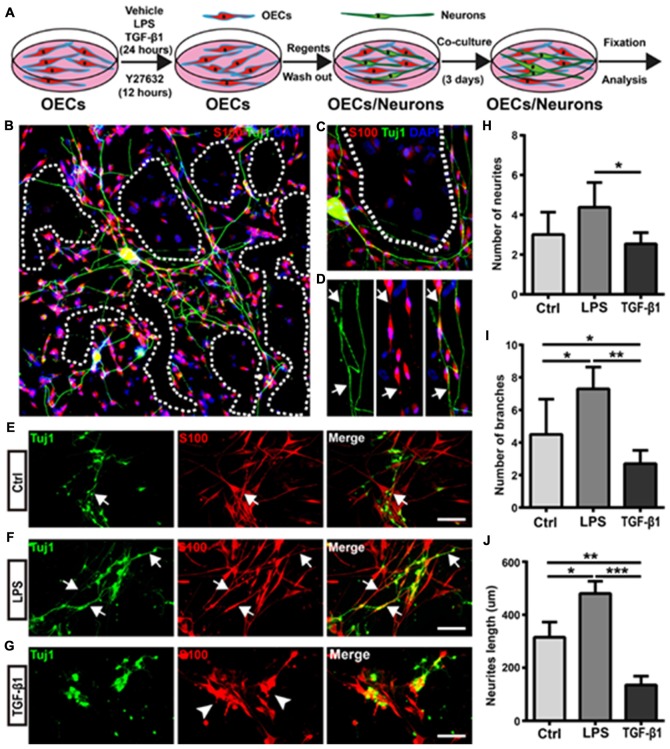
Morphological shift of OECs under pro- and anti-inflammatory conditions affects neurite outgrowth and extension. **(A)** Schematic depiction of OEC/Neuron co-cultures. **(B)** Neuron/OEC co-cultures for 3 days. Dashed white lines show the OEC-free regions. **(C)** Neurons outgrew and extended on OEC surfaces rather than on OEC-free regions. **(D)** Neurites extended along processes of OECs. Arrowheads point to extended neurites along processes of OECs. **(E–G)** Neuron outgrowth on OECs pre-treated with vehicle (Ctrl, **E)**, LPS (10 μg/mL, **F**) or TGF-β1 (10 ng/mL, **G**) for 24 h. Arrows point to extended neurites along processes of OECs. Arrowheads point to OECs that cluster together. **(H, I)** Quantitative assessment of the number of neuronal neurites and branches, respectively, under indicated treatments. **(J)** Quantitative assessment of neurite length under indicated treatments. Data are represented as mean ± SD, *n* = 3 experiments. Scale bar, 100 μm. *P < 0.05, **P < 0.01, ***P < 0.001; one-way ANOVA with Dunnett’s post test.

To further explore the relationship between the morphology and function of OECs, cortical neurons were co-cultured with OECs for 3 days, and neurite outgrowth and extension were examined using Tuj1 staining (Figure 5A). Almost all neurites extended on OEC surfaces rather than on OEC-free regions (Figures 5B,C). In addition, neurites outgrew and extended along processes of OECs (Figure 5D). These results indicated that process-bearing OECs aided and promoted neurite outgrowth and extension, and that the cell contact-mediated effect of OECs was involved.

Next, we evaluated whether morphological changes of OECs under pro- and anti-inflammatory conditions also affected neurite outgrowth and extension. Cortical neurons grew longer and branched more neurites on OECs pre-treated with pro-inflammatory conditions (LPS) for 24 h (Figures 5E,F). Consistent with the above results, neurons outgrew and extended along the processes of OECs (Figure 5F). In contrast, neurons on OECs pre-treated with anti-inflammatory conditions (TGF-β1) showed shorter unbranched neurites (Figure 5G). Additionally, OECs under anti-inflammatory conditions (TGF-β1) grew in clusters and had short or no processes (Figure 5G). Furthermore, neurons on OECs pre-treated with LPS grew with more neurites and branches than those grown on control OECs and OECs pre-treated with TGF-β1 (Figures 5H,I). The total lengths of neurites grown on OECs pre-treated with LPS were longer than those grown on control OECs and OECs pre-treated with TGF-β1 (Figure 5J). These results suggested that OECs pre-treated with pro- and anti-inflammatory conditions differentially affected neurite outgrowth, branch and extension, and morphological shifts of OECs were involved in this neurite outgrowth-promoting effect.

### Morphological Shift of OECs Induced by ROCK Inhibitor Enhanced Neurite Outgrowth and Extension via L1-CAM-Dependent Mechanism

**Figure 6:**
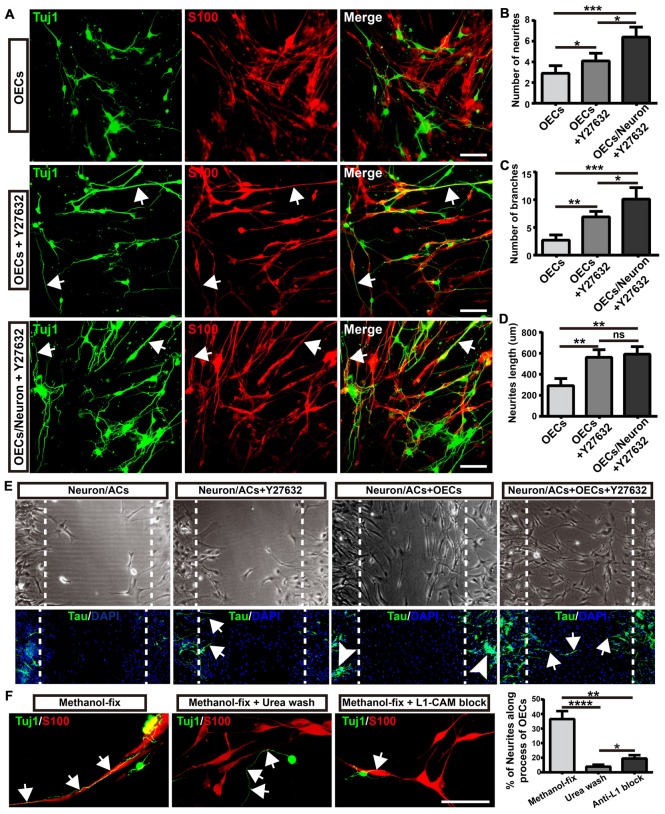
Morphological shift of OECs induced by ROCK inhibitor enhanced neurite outgrowth and extension via L1-CAM-dependent mechanism. **(A)** Neuron outgrowth on OECs under indicated treatments. In OECs group, OECs were co-cultured with neurons. In OECs + Y27632 group, OECs were pre-treated with Y27632 for 12 h to induce towards process-bearing shape and then co-cultured with neurons after washed out Y27632. In OECs/Neuron + Y27632 group, OEC/neuron co-cultures were pre-treated with Y27632 for 12 h and then Y27632 was washed out. After 3 days, cells from all groups had immunocytochemical (ICC) staining performed. **(B,C)** Quantitative assessment of the number of neuronal neurites and branches, respectively, after indicated treatments. **(D)** Quantitative assessment of neurite length under indicated treatments. **(E)** The neurite outgrowth and extension after indicated treatments using *in vitro* scratch assay. Arrows point to extended neurites across the scratched region. Arrowheads point to clustered neurites. Dashed white lines showed the scratched regions. **(F)** OECs were treated with Y27632 for 12 h and fixed by methanol to eliminate the factors secreted by OECs. Methanol-fixed OECs were washed with urea (8 mol/L) for 30 min or blocked by L1-CAM antibody (10 μg/mL) for 4 h. Primary neurons were co-cultured with these methanol-fixed OECs for 3 days and subsequently performed ICC staining to examine neurite outgrowth and extension. Scale bar, 100 μm. Data are represented as mean ± SD, *n* = 3 experiments. *P < 0.05, **P < 0.01, ***P < 0.001, ****P < 0.0001; ns, not significant; one-way ANOVA with Dunnett’s post test.

As the ROCK/F-actin pathway mediated the morphological changes of OECs, we used ROCK inhibitor Y27632 to induce OECs towards a process-bearing shape, and examined neurite outgrowth and extension. OECs were pre-treated with Y27632 for 12 h to induce OECs towards a process-bearing shape, and then cortical neurons were co-cultured with OECs (OECs + Y27632 group). Alternatively, cortical neurons were co-cultured with OECs and the OEC/neuron co-cultures were pre-treated with Y27632 for 12 h (OECs/Neuron + Y27632 group). OEC/neuron co-cultures without Y27632 treatment (OECs group) were defined as the control. Compared with the OECs group, cortical neurons grew with more neurites and branches and extended longer neurites in the OECs + Y27632 group (Figures 6A–D). In addition, adenylate cyclase agonist forskolin, which also induced OECs towards a process-bearing shape, also promoted neurite extension along processes of OECs (data not shown). Strikingly, cortical neurons in the OECs/Neuron + Y27632 group grew with more neurites and branches than in OECs + Y27632 group (Figures 6A–C). Furthermore, a combination of OECs and Y27632 promoted neurites to extend across injured regions using an *in vitro* scratch assay (Figure 6E). All these results suggested that ROCK inhibitor Y27632 induced process-bearing OECs and enhanced neurite outgrowth and extension.

As neurites grew out and extended along the processes of OECs, we wondered whether direct cellular contact with OECs and ECM or adhesion molecules of OECs played key roles in the enhanced neurite outgrowth. To test this hypothesis, we fixed OECs with methanol to preserve their structure (ECM and adhesion molecules) and to eliminate the effect of the factors secreted by OECs. We found that neurites still grew out and extended along the processes of methanol-fixed OECs, indicating that direct cellular contact but not the factors secreted by OECs played a role in the outgrowth of neurites along the processes of OECs (Figure 6F). When the ECM and adhesion molecules of OECs were extracted and disrupted with urea, neurites grew out and extended randomly rather than along the processes of OECs. Furthermore, consistent with a previous study (Witheford et al., 2013), a block of neural adhesion molecule L1-CAM with antibody reduced the extension of neurites along the processes of OECs (Figure 6F). Together, these results suggested that process-bearing OECs induced by ROCK inhibitor Y27632 enhanced neurite outgrowth and extension via cellular contact and L1-CAM-dependent mechanisms.

### YAP Involved in Morphological Shift and Enhanced Neurite Outgrowth-Promoting Property of OECs

**Figure 7:**
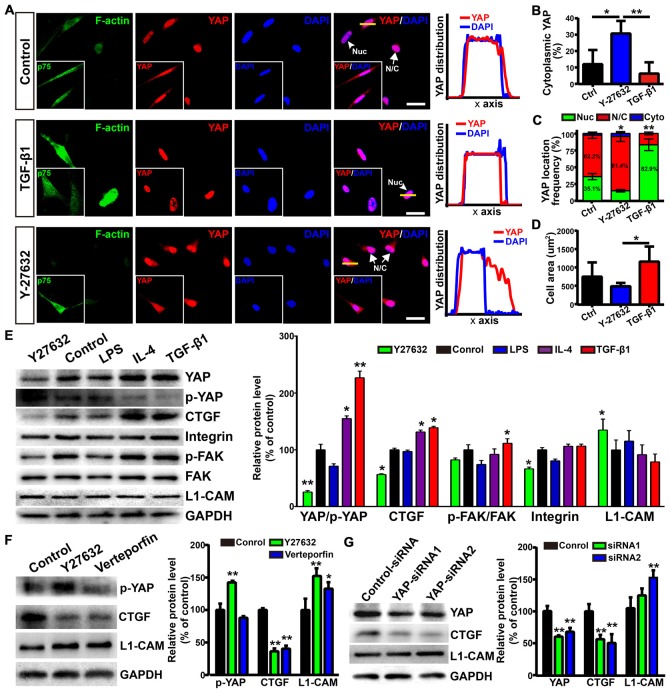
Yes-associated protein (YAP) involved in morphological shift and enhanced neurite outgrowth-promoting property of OECs. **(A)** Images showed the effects of F-actin stress fibers and cell morphology on YAP distribution. The inserts showed p75NTR-positive OECs and the distribution of YAP. Scale bar, 50 μm. **(B)** Quantitative assessment of cytoplasmic YAP under indicated treatments by mean fluorescence intensity. **(C)** The distribution of YAP under indicated treatments. Nuc, nuclear YAP; N/C, nuclear and cytoplasmic YAP; Cyto, cytoplasmic YAP. **(D)** Cellular area of OECs under indicated treatments. **(E)** Protein analysis of the indicated proteins in OECs under indicated treatments. p-YAP, phosphorylation of YAP at Ser127; p-FAK, phosphorylation of FAK at Tyr397. **(F)** OECs were treated with vehicle (0.1% DMSO), Y27632 (10 μM) or verteporfin (5 μM) for 24 h and performed WB analysis. **(G)** OECs were treated with YAP-siRNA or control-siRNA for 72 h and performed WB analysis. All quantitative assessments of WB were normalized by GAPDH and compared to Control. Data are represented as mean ± SD, *n* = 3 experiments. *P < 0.05, **P < 0.01; one-way ANOVA with Dunnett’s post test **(B,D–G)**; χ^2^ (and Fisher’s exact) test **(C)**, comparison with control.

The activity of transcriptional co-activator YAP is correlated to the stability of the actin cytoskeleton and cell tension, which were controlled by Rho GTPase pathways and myosin II (Wada et al., 2011; Nardone et al., 2017). Thus, we wondered whether YAP played a role in morphological shift and the enhanced neurite outgrowth-promoting property of OECs induced by ROCK inhibition. YAP was mostly localized in the nuclei of flattened OECs and in the nuclei and cytoplasm of process-bearing OECs (Figure 7A). Consistent with this, TGF-β1 treatment shifted OECs towards a flattened shape with a larger cellular area, formed more stress fibers and resulted in nuclear YAP accumulation. In contrast, ROCK inhibitor Y27632 shifted OECs towards a process-bearing shape with a smaller cellular area, inhibited the formation of stress fibers and excluded nuclear YAP to cytoplasm (Figures 7A–D). Consistent with YAP distribution, TGF-β1 and IL-4 treatments increased protein levels of YAP and decreased the phosphorylation of YAP at Ser127. Accordingly, the expression of CTGF, a well-characterized target gene of the YAP-TEAD complex, was dramatically up-regulated, indicating that TGF-β1 and IL-4 activated YAP (Figure 7E). In contrast, Y27632 and LPS treatments increased the phosphorylation of YAP at Ser127 and down-regulated the expression of CTGF, indicating that Y27632 and LPS inhibited YAP (Figure 7E). Furthermore, the increased protein levels of integrin and FAK autophosphorylation at the Tyr397 site were found in TGF-β1 and IL-4-treated OECs, indicating activated integrin-focal adhesion (FA) signaling and resultant cytoskeleton stability (Figure 7E). In contrast, a marked reduction in the protein levels of integrin and FAK autophosphorylation were found in Y27632 and LPS-treated OECs, indicating inactivated integrin-FA signaling and disrupted cytoskeleton (Figure 7E). Thus, these results indicated that stress fibers consisting of F-actin mediated the morphological shift of OECs and regulated YAP distribution.

As shown above, L1-CAM was involved in the enhanced neurite outgrowth-promoting property of OECs. Interestingly, Y27632 treatment inhibited YAP activity and increased the protein levels of L1-CAM of OECs (Figure 7E). Similarly, verteporfin, a YAP-TEAD inhibitor, also increased the L1-CAM protein level of OECs (Figure 7F). To further validate the role of YAP in the regulation of L1-CAM, we used siRNA to downregulate YAP protein levels endogenously. YAP-siRNA transfection decreased the YAP protein level of OECs and upregulated the L1-CAM protein level of OECs (Figure 7G). Collectively, these results suggested that YAP signaling downstream of the ROCK pathway mediated morphological shift and enhanced the neurite outgrowth-promoting properties of OECs through upregulating L1-CAM.

## Discussion

**Figure 8:**
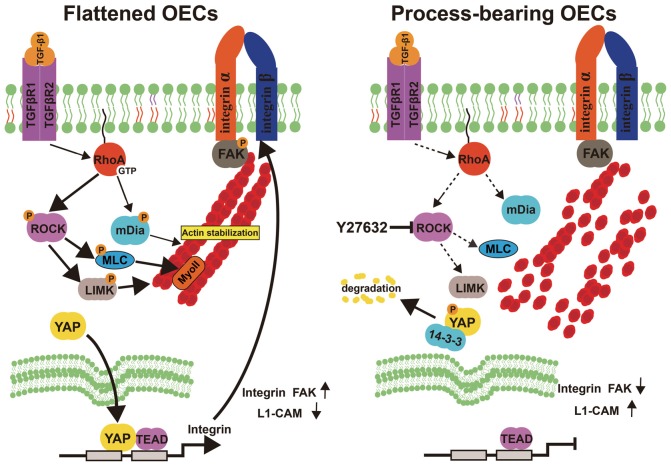
Model proposing ROCK inhibition mediates morphological shift and neurite outgrowth-promoting property of OECs. Left: Activated RhoA/ROCK pathway maintains the integrity of F-actin fiber cytoskeleton, which triggers YAP nuclear shuttling. Nuclear YAP upregulates integrin, activates integrin-focal adhesion (FA) signaling and results in flattened shape of OECs. Right: inhibition of ROCK pathway disrupts F-actin fiber cytoskeleton, which excludes nuclear YAP to cytoplasm. YAP cytoplasmic shuttling shifts OECs towards process-bearing shape, upregulates L1-CAM and enhances neurite outgrowth-promoting property of OECs.

OEC transplantation is intensively regarded as a promising therapeutic strategy for CNS injuries and neurodegenerative diseases. However, different OEC subpopulations showed biological differences and affected the outcomes following OEC transplantation. Herein, we showed that OECs displayed a process-bearing shape under pro-inflammatory conditions (LPS) and a flattened shape under anti-inflammatory conditions (IL-4 and TGF-β1). Functionally, process-bearing OECs under pro-inflammatory conditions showed increased cellular activity and a faster migratory rate than flattened OECs under anti-inflammatory conditions and promoted neurite outgrowth and extension. More importantly, morphological shift of OECs induced by ROCK inhibitor Y27632 enhanced neurite outgrowth and extension via YAP-dependent upregulation of L1-CAM (Figure 8). Because of the shown efficacy of ROCK inhibitors in animal models of spinal-cord injury and demyelinating diseases, our results highlighted the potential combination of OEC transplantation and ROCK inhibitors in therapy of CNS injuries or neurodegenerative disorders.

From a morphological point of view, OECs have two subpopulations: process-bearing and flattened OECs. OECs were able to switch spontaneously between process-bearing and flattened shapes and underwent rapid and reversible morphological changes depending on environmental stimuli (Vincent et al., 2003; Huang et al., 2008). Herein, we showed that OECs displayed a process-bearing shape under pro-inflammatory conditions (LPS) and a flattened shape under anti-inflammatory conditions (IL-4 and TGF-β1). In addition, process-bearing OECs rapidly shifted to a flattened shape by replacing LPS with TGF-β1. However, flattened OECs induced by anti-inflammatory conditions (IL-4 and TGF-β1) were hardly able to be reversed back to process-bearing shape by replacement with LPS. Previous studies showed that, when dBcAMP was replaced with ET-1, process-bearing OECs returned to a flattened morphology within 1 h. Conversely, when ET-1 was replaced with dB-cAMP, flattened OECs returned to process-bearing morphology within 3 h (Vincent et al., 2003). On the basis of our present data as well as previous reports, it seems to be that flattened OECs are of a more stable state than process-bearing OECs. The Rho/ROCK signaling pathway played important roles in the cell shape of many cell types. The RhoA/ROCK/Myosin pathway also mediates the morphological switches of OECs through cytoskeletal re-arrangements, including stress fibers and FAs and local disruption of active RhoA distributions promotes process outgrowth and leads to morphological changes from a flattened shape into a process-bearing shape (Huang et al., 2011b). Consistent with these previous findings, we have shown that the ROCK/F-actin signaling pathway also mediated morphological changes of OECs under pro- and anti-inflammatory conditions. Activation of the RhoA/ROCK pathway results in myosin contractility and F-actin stabilization, which play important roles in the formation of stress fibers (Burridge and Wennerberg, 2004). Conversely, inactivation of RhoA/ROCK increases the amount of dephosphorylated profilin IIa, resulting in F-actin destabilization and sprout formation (Hall and Lalli, 2010). These morphological regulation mechanisms, which involved multiple small GTPases and downstream effectors, may contribute to the partially reversible switches between process-bearing and flattened OECs.

The phenomenon whereby cell shape changes are associated with distinct functional states has been found in endothelial cells, stem cells, vascular smooth muscle cells and macrophages (McBeath et al., 2004; Alford et al., 2011; Versaevel et al., 2012; McWhorter et al., 2013). Strikingly, pro-inflammatory and anti-inflammatory cytokines also induced different cell shapes in macrophages (McWhorter et al., 2013). Adding LPS and IFN-γ, which induce M1 polarization, caused macrophages to change into a round, pancake-like shape. In contrast, adding IL-4 and IL-13, which induce M2 polarization, led to an increased degree of cell elongation. Similarly, shape changes of macrophages were also dependent on actin polymerization and cytoskeletal contractility. Interestingly, IL-4 treatment induced a process-bearing shape in macrophages, but a flattened shape in OECs. The molecular mechanism that led IL-4 to induce different cell shapes for macrophages and OECs needs further investigation. In addition, our previous study showed that TGF-β1 treatment induced a flattened shape, increased phagocytic activities of OECs and enhanced neuronal survival (Li et al., 2017). In the present study, we found that process-bearing OECs under pro-inflammatory conditions (LPS) showed a faster migratory rate over flattened OECs and promoted neurite outgrowth and extension. Several studies showed that the process-bearing OECs myelinated axons in lesion environments (Devon and Doucette, 1992; Ramón-Cueto et al., 1993). It seems that flattened OECs are supportive cells for neuronal survival, and process-bearing OECs provide permissive substrates, myelinate axons and promote neurite outgrowth and extension. Thus, our present data as well as previous reports support the notion that cellular morphology is associated with the functional phenotype of OECs. However, although two subpopulations of OECs have been identified in the ONL according to their expressions of different cell markers (Vincent et al., 2005), there is no data to show that these *in situ* OECs can be morphologically divided into flattened and process-bearing types. Further studies might explore and find a particular morphology of *in situ* or cultured OECs that is associated with a regeneration-promoting phenotype.

OECs migrate ahead of primary olfactory axons during development and regeneration. Moreover, outgrowth of olfactory axons is dependent on the motility of underlying OECs and stimulation of the motility of OECs enhances axon extension and growth cone activity *in vitro* (Windus et al., 2011). Furthermore, the migratory property of OECs is considered to contribute to the “repair” abilities following OEC transplantation. OECs migrate and associate with extending axons and are proposed to provide permissive substrates for neuronal bridging beyond the injury site. However, controversial results regarding the migration of transplanted OECs have been reported after transplantation in lesioned CNS. Some studies showed that OECs readily coexist with reactive astrocytes and reactive astrocytes in glial scars released TNF-α to attract transplanted OECs migrating from the injection site into the lesion center (Ramón-Cueto and Nieto-Sampedro, 1994; Lakatos et al., 2003; Su et al., 2009). Other studies demonstrated that transplanted OECs migrated preferentially in the opposite direction to the regenerating axon target, and the motility of transplanted OECs in damaged CNS was limited (Gudiño-Cabrera et al., 2000; Lu et al., 2006). Indeed, transplanted OECs are confronted with a complex and changing environment in the glial scar and adjacent spinal cord regions, where a huge variety of growth-inhibitory molecules and myelin debris is located. Several myelin-associated inhibitors (MAIs), including Nogo-A, the myelin-associated glycoprotein (MAG) and the oligodendrocyte-myelin glycoprotein (OMgp), block the migration of OECs and axon regeneration by acting through the Nogo receptor complex. In this study, we showed that process-bearing OECs displayed a faster migratory rate than flattened OECs. Our previous study showed that TGF-β1 induced by neuronal debris shifted OECs towards a flattened shape and increased phagocytic activities of OECs through upregulating the integrin/MFG-E8 pathway (Li et al., 2017). All these results support the hypothesis that myelin debris in the injured regions induces secretion of TGF-β1, which shifts OECs towards a flattened shape to enhance the phagocytic clearness of myelin debris and decreases the migratory ability of OECs. Interestingly, integrins were reported to be receptors for the N-terminal domain of Nogo-A, which inhibited cell adhesion and axonal outgrowth (Hu and Strittmatter, 2008). Further studies will address whether integrins integrate signals of OECs emanating from phagocytosis and migration and regulate the morphological shift of OECs, or inversely.

Rho GTPases, including Rho, Rac and Cdc42, play a major role in controlling the assembly, disassembly, and dynamic rearrangements of the actin and microtubule cytoskeletons (Colicelli, 2004). Thus, it is not surprising that they play crucial roles in the growth and branching of neuronal axons (Hall and Lalli, 2010). A variety of studies suggests that Rho and its downstream effector ROCK negatively regulate the early steps of axonal outgrowth in cultured neurons, and inhibition of the Rho/ROCK pathway promotes the outgrowth of neurites. In contrast, Rac and Cdc42 are positive regulators that promote neurite extension (Kranenburg et al., 1999; Li et al., 2002; Govek et al., 2005). Studies reveal that not only the expression of the RhoA pathway is induced by injury, but also its activation is stimulated by MAIs, which accounts for an irreversible arrest of neurite outgrowth (Dubreuil et al., 2003; Brabeck et al., 2004; Madura et al., 2004). In addition, the persistent presence of neurite-growth inhibitors at or around the lesion site results in a strong and persistent barrier for regenerative neurite outgrowth through Rho/ROCK activation (Mueller et al., 2005). Thus, ROCK inhibitors might help injured neuronal fibers to grow or sprout beyond this regeneration-inhibiting tissue. Previous studies demonstrated that ROCK inhibition enhanced neuronal fiber growth beyond the lesion site and local application of Y27632 improved functional recovery in transection spinal-cord injuries (Fournier et al., 2003; Sung et al., 2003; Mueller et al., 2005). In this study, we found that ROCK inhibitor Y27632 induced process-bearing OECs and promoted the outgrowth and branching of neurites through YAP-dependent upregulation of L1-CAM. Cell adhesion molecules of the immunoglobulin superfamily (IgSF), including the neural cell adhesion molecule (NCAM) and members of the L1 family of neuronal cell adhesion molecules, play important roles in the developing nervous system by regulating the formation, growth and branching of neurites (Schmid and Maness, 2008; Leshchyns’ka and Sytnyk, 2016). AAV-mediated L1-CAM over-expression appeared to favorably modify the local environment in the injured spinal cord and promoted regeneration (Chen et al., 2007). Trimebutine, a small molecule mimetic agonist of L1-CAM, was also shown to contribute to functional recovery after spinal cord injury (Xu et al., 2017). In OECs, L1-CAM of OEC stimulated corticospinal tract (CST) outgrowth even when inhibition is induced by MAG (Witheford et al., 2013). Interestingly, the intracellular domains of IgSF cell adhesion molecules interact with the components of the cytoskeleton; in turn, the cytoskeleton plays an important role in regulating the functions of IgSF cell adhesion molecules (Leshchyns’ka and Sytnyk, 2016). Whether the upregulated L1-CAM affects morphological changes of OECs via a positive feedback mechanism to promote neurite outgrowth needs further study. Together, the combination of OECs and small-molecule ROCK inhibitors might have a potentially significant benefit for stimulating neuroregeneration in spinally injured patients. In addition, forskolin, which also affects the shape of OECs, enhanced neurite outgrowth and extension (data not shown). This might explain contradictory outcomes following OEC transplantation as due whether forskolin was used or not in OEC culture medium.

In summary, our data demonstrated that the ROCK/F-actin pathway was involved in reversible morphological changes of OECs under pro-inflammatory and anti-inflammatory conditions. Furthermore, the use of ROCK inhibitor Y27632 enhanced the neurite outgrowth-promoting property of OECs via YAP-dependent upregulation of L1-CAM, providing a potential combination of OECs and small-molecule ROCK inhibitors for the treatments of CNS injuries. Future experiments will be directed to explore whether these OEC phenotypes affect functional axon regeneration of transplanted OECs for CNS injuries.

## Author Contributions

YL and SH: conception and design, manuscript writing and final approval of manuscript. YL, SH and TZ: conducted OEC cell experiment and data collection. YF, XG and QT: neuron experiment conduction and data collection. HX: conception and design, manuscript review and revision and final approval of manuscript. All authors critiqued the manuscript and approved its submission.

## Conflict of Interest Statement

The authors declare that the research was conducted in the absence of any commercial or financial relationships that could be construed as a potential conflict of interest.
