# Supplementary material for: ROCK Inhibitor Y27632 Induced Morphological Shift and Enhanced Neurite Outgrowth-Promoting Property of Olfactory Ensheathing Cells via YAP-Dependent Up-Regulation of L1-CAM
Source: Front Cell Neurosci. 2018 Dec 11;12:489. doi: 10.3389/fncel.2018.00489 (PMC6297255; doi:10.3389/fncel.2018.00489)
Supplement: Supplementary file 1 [file Data_Sheet_1.doc]

**Supplementary Material:**

**Methods:**

**1. Cell Count**

To determine OECs number and the ratios of BrdU/Ki67 positive OECs, OECs were counted as following. Briefly, from each glass slide, randomly chosen 4 fields were photographed through the fluorescent microscope. All OECs (DAPI staining) and BrdU/Ki67 positive OECs were counted. Half of cell count was performed by a person blinded to the experimental setting. The ratios of BrdU/Ki67 positive OECs were calculated. In all analysis, data represent the mean±S.D. of at least 3 independent experiments.

**2. Cell Counting Kits-8 (CCK-8) Assay**

CCK-8 assay was performed according to the instructions of the manufacturer (Donjidon Lab, Japan). Briefly, OECs were seeded in 96-well plates and cultured for 2 days, which reached about 60% confluence. OECs were further cultured under control, pro-inflammatory (LPS) or anti-inflammatory (IL-4 and TGF-β1) conditions for 48 hours. 10μL CCK-8 assay CellTiter96 Aqueous One Solution reagent (Donjidon Lab) was added into 96-well plates. After 1 hour, the cellular metabolic activity of OECs was determined by colorimetric assay with absorbance at 450nm and 670nm measured by Microplate Reader (Varioskan Flash, Thermo Scientific). The difference of absorbance at 450nm and 670nm was analyzed and Blank group (medium without OECs) was used to reduce systematic errors. Data represent the mean±S.D. of 3 independent experiments.

**Supplementary Figure Legends:**

**Supplementary Fig. 1: Morphological changes of OECs on different substrates**. (**a**) Morphological changes of OECs (red, S100) on gelatin and laminin after treatments with 10μg/mL LPS, 10ng/mL IL-4, and 10ng/mL TGF-β1 for 24 hours. Scale bar, 50µm. (**b-d**) Average process length, circularity and percentage of flattened and process-bearing OECs for LPS, IL-4 and TGF-β1-treated purified OECs on gelatin. (**e-f**) Average process length, circularity and percentage of flattened and process-bearing OECs for LPS, IL-4 and TGF-β1-treated purified OECs on laminin. Values in the histogram represent the counted number of OECs (**b**, **c**, **e**, **f**) or percentage of process-bearing OECs (**d**, **g**). Data are represented as mean ± SD, n = 3 experiments. ***** P < 0.05, ****** P < 0.01, ******* P < 0.001, ******** P < 0.0001; one-way ANOVA with Dunnett’s post test (**b**, **c**, **e**, **f**) and **χ**^2^ (and Fisher’s exact) test (**d**, **g**), comparison with control.

**Supplementary Fig. 2: The effects of pro- and anti-inflammatory conditions on OECs proliferation.** (**a**) Ki67 positive OECs after treatments with 10μg/mL LPS, 10ng/mL IL-4, and 10ng/mL TGF-β1 for 24 hours. (**b**) A scatter dot diagram shows the ratio of Ki67 positive OECs under pro- or anti-inflammatory conditions. (**c**) Immunostaining images of BrdU incorporation into OECs under pro- or anti-inflammatory conditions at 24 hours. (**d**) A scatter dot diagram shows the ratio of BrdU positive OECs under pro- or anti-inflammatory conditions. (**e**) The cell number of OECs under pro- or anti-inflammatory conditions per field (100x). (**f**) Quantification of the cellular metabolic activity of OECs using CCK-8 assay. Scale bar, 50µm. Data are represented as mean ± SD, n = 3 experiments. ***** P < 0.05; ns, not significantly different; one-way ANOVA with Dunnett’s post test.
