# Supplementary figures and images for: ROCK Inhibitor Y27632 Induced Morphological Shift and Enhanced Neurite Outgrowth-Promoting Property of Olfactory Ensheathing Cells via YAP-Dependent Up-Regulation of L1-CAM
Source: Front Cell Neurosci. 2018 Dec 11;12:489. doi: 10.3389/fncel.2018.00489 (PMC6297255; doi:10.3389/fncel.2018.00489)

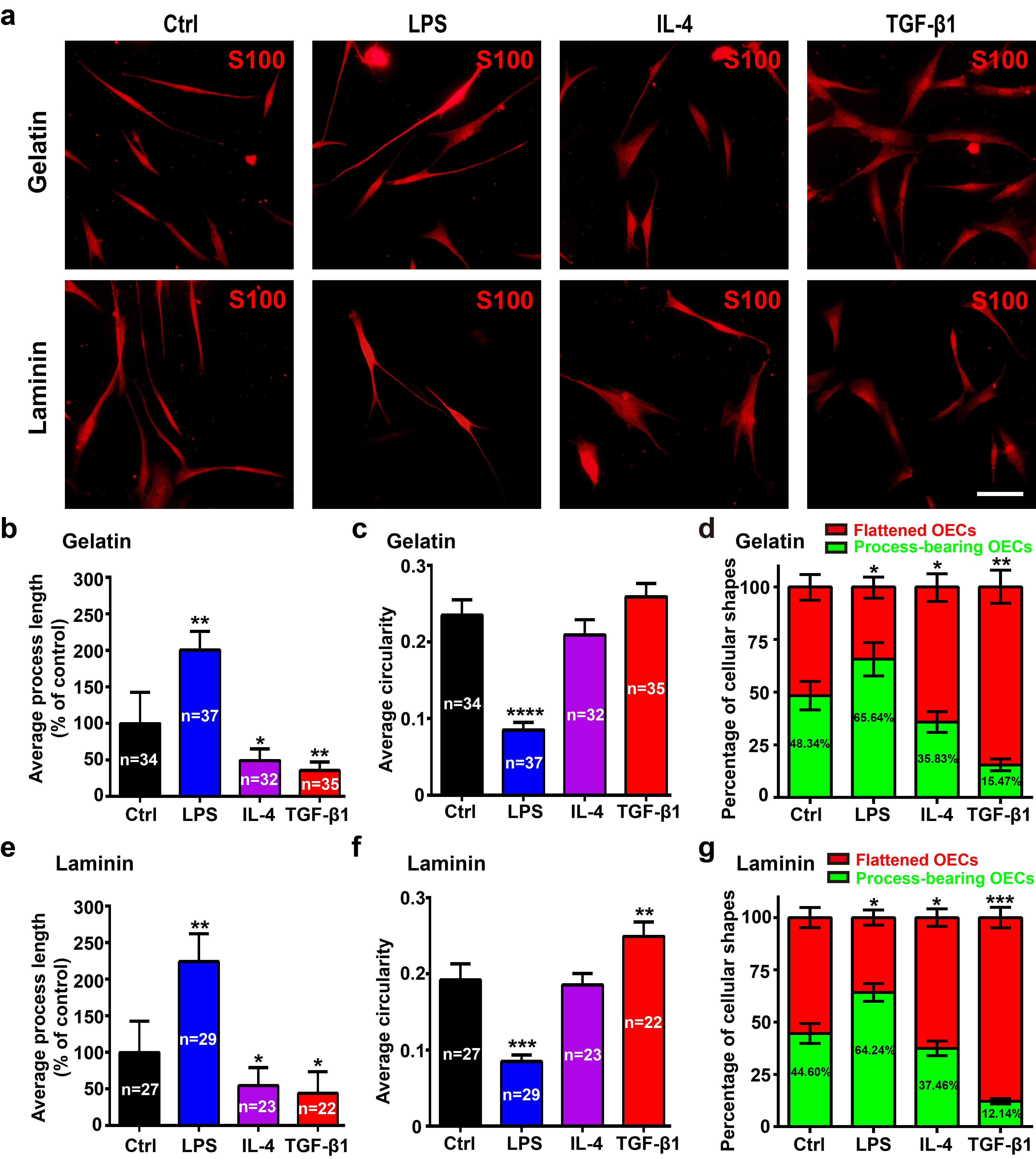

Supplement: Supplementary file 2 [file Image_1.JPEG]

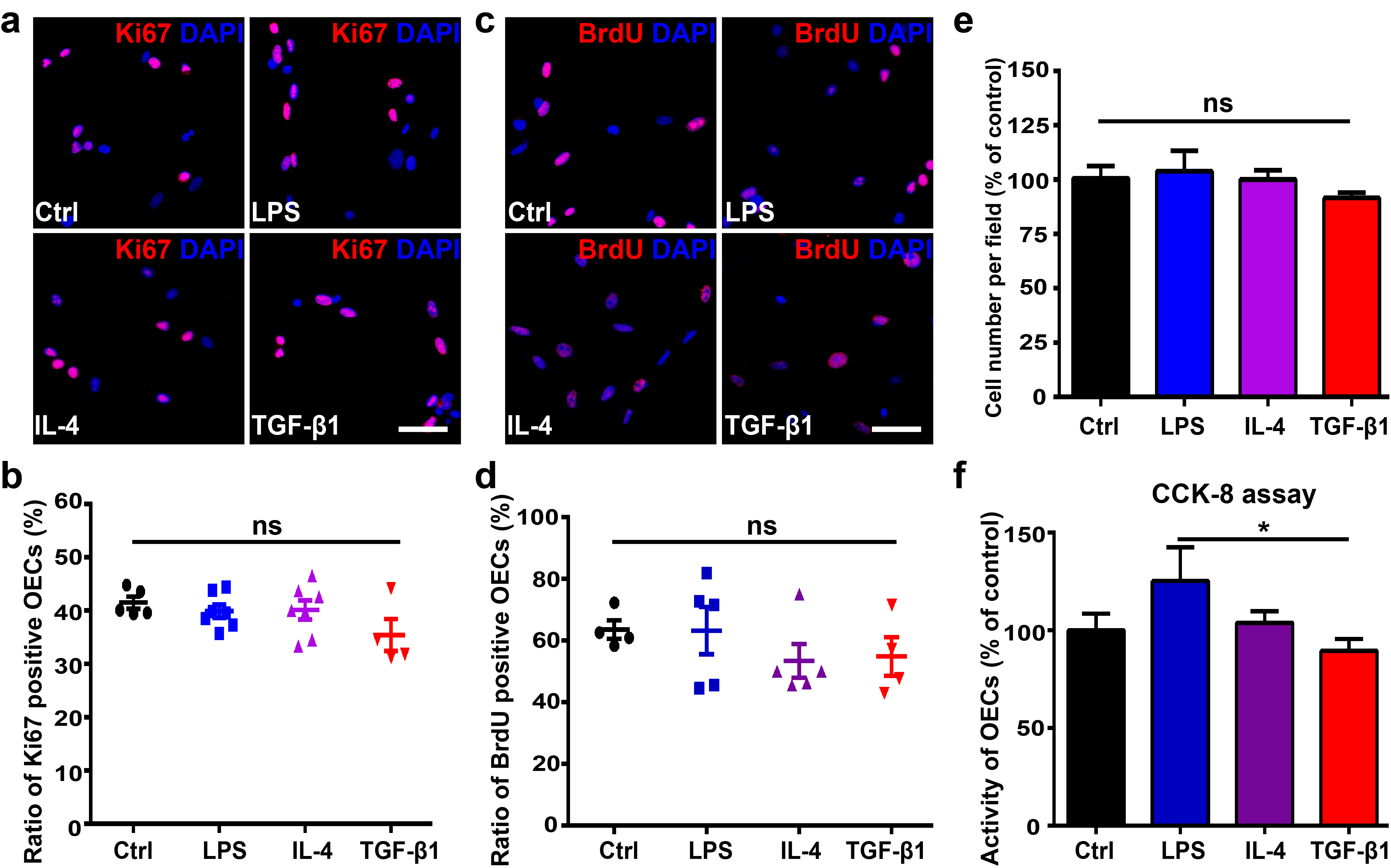

Supplement: Supplementary file 3 [file Image_2.JPEG]
